# Factors associated with vitamin D deficiency among patients with musculoskeletal disorders seeking physiotherapy intervention: a hospital-based observational study

**DOI:** 10.1186/s12891-022-05774-z

**Published:** 2022-08-30

**Authors:** Mohammad Ali, Zakir Uddin

**Affiliations:** 1 Department of Physiotherapy and Rehabilitation, Uttara Adhunik Medical College and Hospital, Sector-09, Uttara Model Town, Dhaka, 1230 Bangladesh; 2 Low Back Research Team, College of Science, Health & Engineering, La Trobe University, Bundoora, VIC Australia; 3 School of Rehabilitation Sciences, McMaster University, Hamilton, ON L8S 4L8 Canada

**Keywords:** Vitamin D, Musculoskeletal disorders, Physiotherapy, Public health, Low back pain, Sun exposure

## Abstract

**Background:**

A considerable number of studies have suggested that there is a strong correlation between 25-hydroxyvitamin D or vitamin D levels and overall health, with reported impacts ranging from mental health and vital organ health to musculoskeletal health. This study aimed to determine the prevalence of 25-hydroxyvitamin D deficiency and identify its associated factors among patients with musculoskeletal disorders (MSDs) currently seeking medical attention.

**Methods:**

A total of 143 patients with MSDs were randomly selected for blood sample collection to measure serum 25-hydroxyvitamin D levels. Descriptive statistics were used to describe the demographic and clinical characteristics of the study participants. Multiple logistic regression analyses were performed to compute the adjusted odds ratio.

**Results:**

Overall, 53.1% of patients had vitamin D deficiency. Vitamin D deficiency was more prevalent among patients with higher body mass index, a bachelor’s degree, lower sun exposure time, and lower serum calcium levels and those living in an urban setting. The multiple logistic regression model suggested that the duration of weekly sun exposure and living location were the independent predictors of vitamin D deficiency.

**Conclusion:**

It is recommended for patients with MSDs to participate in routine physical exercise, consume calcium- and vitamin D-enriched foods, and have regular sun exposure for minimizing the risk of vitamin D deficiency.

## Background

Vitamin D, or 25-hydroxyvitamin D [25(OH)D], is a fat-soluble essential vitamin that regulates calcium homeostasis and is crucial for human health across all ages [1]. Many foods and dietary supplements contain vitamin D naturally, and it can be produced endogenously through vitamin D synthesis when the skin is exposed to the ultraviolet rays of sunlight [2]. A serum concentration of less than 30 nmol/L (nmol/L = 0.4 ng/mL) of 25(OH)D, which is the best indicator of vitamin D levels, is considered inadequate for the general health and wellbeing of adults [3].

A considerable number of studies have suggested that there is a strong correlation between vitamin D levels and overall health, with reported impacts ranging from mental health and vital organ health to musculoskeletal health [4–10]. A systematic review and meta-analysis of randomized controlled trials concluded that vitamin D supplementation can significantly reduce depression, whereas another review reported vitamin D deficiency to be associated with thyroid disorders [11, 12]. Additional systematic reviews and meta-analyses found significant associations between vitamin D deficiency and arthritis, muscle pain, and chronic widespread pain [13].

Vitamin D deficiency is highly prevalent worldwide, with estimated prevalence of 24%, 37%, and 40% reported in the United States, Canada, and Europe, respectively [14]. A study conducted in Bangladesh found that only 18% of study participants had sufficient serum vitamin D levels [15]. Another study found vitamin D deficiency among 100% of Bangladeshi female garment workers [16]. Regarding South Asian adults, systematic reviews and meta-analysis conducted in 2021 suggested that the highest prevalence of vitamin D deficiency was found in Pakistan (73%; 95% confidence interval [CI], 63–83%), followed by Bangladesh (67%; 95% CI, 50–83%), India (67%; 95% CI, 61–73%), Nepal (57%; 95% CI, 53–60%), and Sri Lanka (48%; 95% CI, 41–55%) [17].

The prevalence of vitamin D deficiency among the general population, community dwellers, elderly individuals, women, and several patient groups has previously been measured [18]. Most studies have been conducted among healthy individuals; however, little is known about the vitamin D levels of patients who are currently seeking medical attention for musculoskeletal disorders (MSDs) in a low-resource developing country, such as Bangladesh. Given the influence of vitamin D on musculoskeletal health, this study aimed to determine the prevalence of vitamin D deficiency and to identify its associated factors in patients seeking physiotherapy intervention for MSDs.

## Methods

### Study participants

In total, 200 patients aged ≥ 18 years with MSDs (lower back, neck, shoulder, or knee pain) were randomly selected from patients seeking physiotherapy treatment at the Department of Physiotherapy and Rehabilitation of Uttara Adhunik Medical College and Hospital and Hasna Hena Pain and Physiotherapy and Public Health Research Center in Dhaka, Bangladesh. Ultimately, 150 patients who provided informed consent to participate in this study were enrolled. The sociodemographic and clinical data of the participants were recorded using a paper-based semi-structured questionnaire. Subsequently, the patients were taken to the Department of Pathology and Biochemistry at Uttara Adhunik Medical College and Hospital for blood sample collection. Finally, 143 patients provided specimens following the standard protocol of the concerned department. We excluded participants aged < 18 years or those who were experiencing pain because of cancer or tuberculosis. Data were collected from May 2020 to February 2021 for this cross-sectional analysis.

### Sociodemographic and clinical data

Sociodemographic data consisted of age, sex, marital status, weight, height, monthly household income in Bangladeshi Taka, occupation, working conditions, education, and residential location (urban/rural). The participants also provided data regarding sun exposure time, physical activity, and smoking habits. Information on previously diagnosed comorbidities, including diabetes, hypertension, and cardiovascular disease, was thoroughly reviewed to record clinical data. Nutrition levels were measured using the Mini Nutritional Assessment Long Form. Individuals were divided into the following three groups: (a) malnourished, (b) at risk of malnutrition, and (c) normal nutritional status [19]. Data regarding chief complaints (lower back/neck/should/knee pain) and pain duration (acute, subacute, and chronic) were also obtained. Pain duration of ≤ 6 weeks, 7–11 weeks, and ≥ 12 weeks were considered acute, subacute, and chronic, respectively [20].

### Laboratory measurements

A chemiluminescence microparticle immunoassay (ARCHITECT i1000SR, USA) was performed to measure the serum concentration of 25(OH)D. The participants were classified based on vitamin D levels as deficient (< 20 ng/mL), insufficient (20–30 ng/mL), or sufficient (30–100 ng/mL) [21]. To estimate hemoglobin levels, a hematology analyzer (Sysmex XN-1000, Japan) was used. Participants with hemoglobin levels between 13 and 18 g/dL were considered normal. Serum calcium levels were measured using the selective electrode technique with automatic correction for pH variation. The reference range used was 8.4–10.2 mg/dL.

### Data analysis

Patients were clustered into two groups according to the serum concentration of 25(OH)D. Patients who had a serum 25(OH)D concentration of < 20 ng/mL were assigned to the vitamin D deficiency group, whereas all other patients were assigned to the “no vitamin D deficiency” group. Descriptive statistics were computed to describe the demographic and clinical characteristics of the study participants. Moreover, χ^2^ tests were conducted to compute vitamin D deficiency proportions and draw comparisons between the groups. Multiple logistic regression analyses were performed to identify predictors of vitamin D deficiency and compute the adjusted odds ratios (AORs) with 95% CIs, considering vitamin D deficiency as a dependent variable and sociodemographic and clinical data as predictor variables for vitamin D deficiency. The regression model included all statistically significant variables associated with vitamin D deficiency in the descriptive analysis and adjusted for age and gender. To ensure that the models adequately fit the data, the Hosmer–Lemeshow goodness-of-fit test was performed (χ^2^ = 4.247; *p* = 0.834). The significance level was set at a *p*-value of < 0.05, and the Statistical Package for the Social Sciences version 22.0 (International Business Machines Corporation) was used for all data analyses.

## Results

### Patient characteristics

Table 1 summarizes patient characteristics and detailed descriptive analysis results. The mean age, body mass index (BMI), duration of sun exposure (h/week), and serum calcium level (mg/dL) of the participants were 49.41 years (standard deviation [SD] ± 13.62), 26.35 (SD ± 3.94), 1.40 h (SD ± 2.39), and 9.50 mg/dL (SD, ± 0.59), respectively. Majority of the participants were women (63.6%), were married (86.7%), were from middle-income families (56.6%), were homemakers (53.1%), were engaged in ordinary work (60.8%), had primary or secondary school education (39.9%), lived in urban areas (74.1%), were nonsmokers (85.3%), and did not perform regular physical exercise (67.1%). Furthermore, 25.4%, 44.1%, 7.7%, and 49% of the participants had diabetes, had hypertension, had cardiac conditions, or were at risk of malnourishment, respectively. However, a majority of chief complaints were lower back pain (50.3%), and 74.1% of the participants were experiencing chronic pain conditions.

**Table 1:** Distribution of vitamin D deficiency according to sociodemographic and clinical data

Variable	All (%) or mean (± SD)	Vitamin D deficiency		*P*-value
		Yes	No	
Overall	143 (100%)	76 (53.1)	67 (46.9)	-
Age (year)	49.41 (13.62)	49.16 (13.05)	53.28 (13.49)	0.065
*Sex*				0.569
Female	91 (63.6)	50 (54.9)	41 (45.1)	
Male	52 (36.4)	26 (50)	26 (50)	
*Marital status*				0.062
Married	124 (86.7)	71 (57.3)	53 (42.7)	
Unmarried	2 (1.4)	0 (0.00)	2 (100)	
Divorce	2 (1.4)	1 (50)	1 (50)	
Widow	15 (10.5)	4 (26.7)	11 (73.3)	
BMI	26.35 (3.94)	26.93 (3.84)	25.67 (3.98)	**0.050**
*Household income (BDT)*				0.324
< 15,000	24 (16.8)	11 (45.8)	13 (54.2)	
15,000–30,000	81 (56.6)	41 (50.6)	40 (49.4)	
> 30,000	38 (26.6)	24 (63.2)	14 (36.8)	
*Occupation*				0.130
Home maker	76 (53.1)	41 (53.9)	35 (46.1)	
Service	37 (25.9)	24 (64.9)	13 (35.1)	
Business & Others	17 (11.9)	7 (41.2)	10 (58.8)	
Retired	13 (9.1)	4 (30.8)	9 (69.2)	
*Working condition*				0.069
Monotonous	37 (25.9)	25 (67.6)	12 (32.4)	
Heavy weightlifting	19 (13.3)	7 (36.8)	12 (63.2)	
Ordinary	87 (60.8)	44 (50.6)	43 (49.4)	
*Education*				**0.027**
Masters and above	24 (16.8)	16 (66.7)	8 (33.3)	
Bachelor	25 (17.5)	17 (68.0)	8 (32.0)	
Higher secondary school	22 (15.4)	12 (54.5)	10 (45.5)	
Primary to secondary school	57 (39.9)	28 (49.1)	29 (50.9)	
No school education	15 (10.5)	3 (20.0)	12 (80.0)	
*Living location*				** < 0.001**
Urban	106 (74.1)	69 (65.1)	37 (34.9)	
Rural	37 (25.9)	7 (18.9)	30 (81.1)	
*Crowding*				0.236
≤ 1.5	68 (47.6)	34 (50.0)	34 (50.0)	
1.6–2.0	46 (32.2)	29 (63.0)	17 (37.0)	
≥ 2.1	29 (20.3)	13 (44.8)	16 (55.2)	
*Sun exposure (h/week)*	1.40 (± 2.39)	0.37 (1.07)	2.58 (2.90)	** < 0.001**
*Physical Activity*				0.363
No exercise	96 (67.1)	55 (57.3)	41 (42.7)	
15–30 min	25 (17.5)	11 (44.0)	14 (56.0)	
> 30 min	22 (15.4)	10 (45.5)	12 (54.5)	
*Smoking habit*				0.601
No	122 (85.3)	64 (52.5)	58 (47.5)	
Yes	14 (9.8)	7 (50.0)	7 (50.0)	
Quitted	7 (4.9)	5 (71.4)	2 (28.6)	
*Diabetes*				0.877
No	108 (75.5)	57 (52.8)	51 (47.2)	
Yes	35 (24.5)	19 (54.3)	16 (45.7)	
*Hypertension*				0.130
No	80 (55.9)	47 (58.8)	33 (41.3)	
Yes	63 (44.1)	29 (46.0)	34 (54.0)	
*Cardiac condition*				0.595
No	132 (92.3)	71 (53.8)	61 (46.2)	
Yes	11 (7.7)	5 (45.5)	6 (54.5)	
*Serum Calcium level (mg/dl)*	9.50 (± 0.59)	9.39 (0.57)	9.60 (0.61)	**0.037**
*Hemoglobin level (g/dl)*				
Female	11.55 (± 1.27)	11.57 (1.32)	11.54 (0.93)	0.894
Male	13.83 (± 1.28)	13.67 (1.22)	13.97 (1.34)	0.410
*Nutrition level*				0.537
Malnourished	8 (5.6)	5 (62.5)	3 (37.5)	
Risk of malnourishment	70 (49.0)	34 (48.6)	36 (51.4)	
Nourished	65 (45.5)	37 (56.9)	28 (43.1)	
*X-ray findings*				0.384
No degenerative change	21 (14.7)	13 (61.9)	8 (38.1)	
Degenerative change	122 (85.3)	63 (51.6)	59 (48.4)	
*Main complaints*				0.166
Low back pain	72 (50.3)	38 (52.8)	34 (47.2)	
Neck pain	31 (21.7)	21 (67.7)	10 (32.3)	
Shoulder pain	16 (11.2)	8 (50.0)	8 (50.0)	
Knee pain	24 (16.8)	9 (37.5)	15 (62.5)	
*Pain duration*				0.811
Acute	25 (17.5)	12 (48.0)	13 (52.0)	
Sub-acute	12 (8.4)	6 (50.0)	6 (50.0)	
Chronic	106 (74.1)	58 (54.7)	48 (45.3)	

Bold *p*-values are significant at a 5% significance level

### Descriptive analysis

**Fig. 1:**
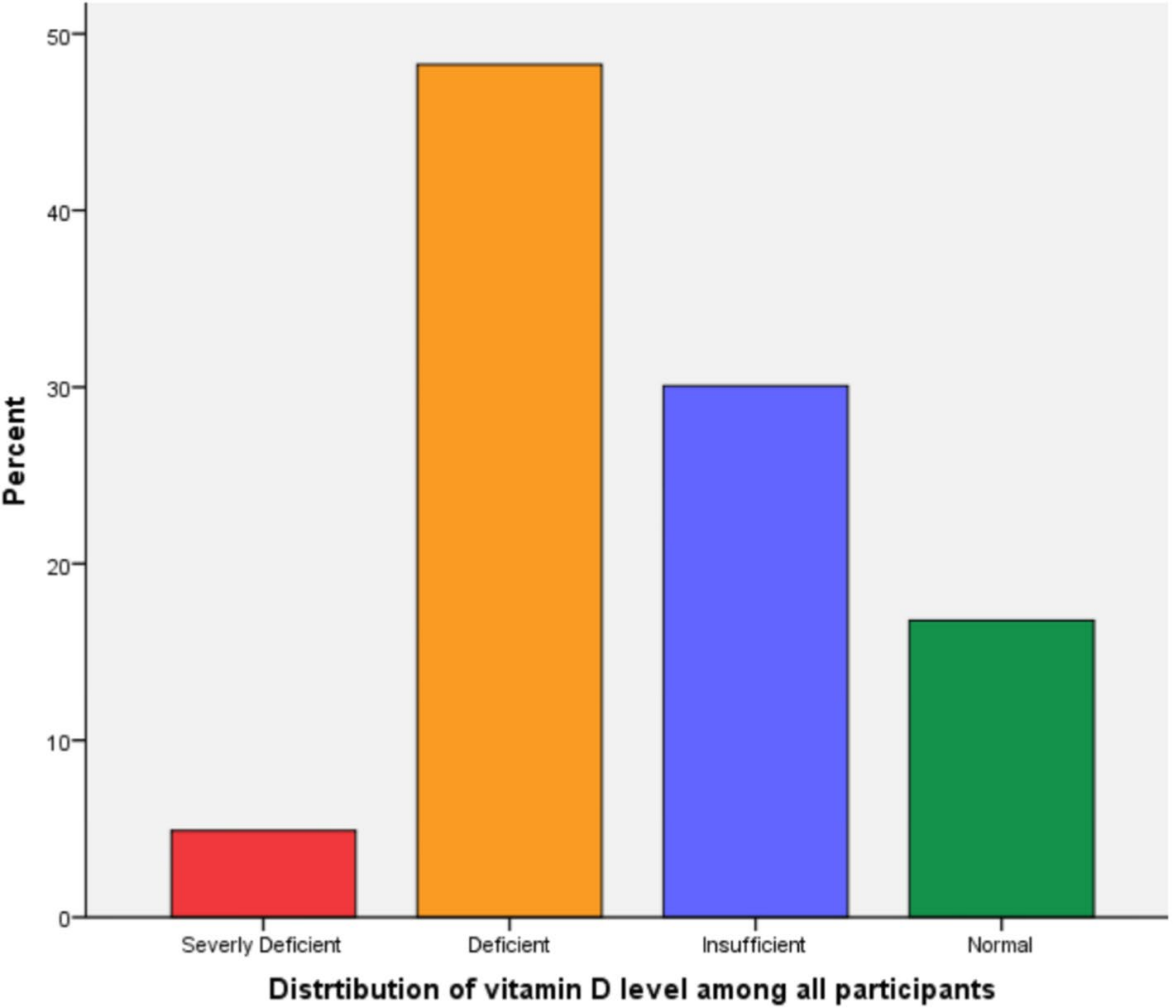
25-hydroxyvitamin D deficiency in patients with musculoskeletal disorders

Overall, 53.1% of the participants had vitamin D deficiency. The mean vitamin D level was 21.82 nmol/L (SD ± 9.83; range, 7.92–66.00 nmol/L). Vitamin D deficiency was more prevalent among patients with higher BMI (*p* = 0.050), a bachelor’s degree (68.0%, *p* = 0.027), lower sun exposure time (*p* < 0.001), and lower serum calcium levels (*p* = 0.037) and those living in an urban setting (74.1%, *p* < 0.001) (Table 1). In contrast, Fig. 1 shows the distribution of vitamin D levels among the study participants. Only 16.8% of the participants had sufficient vitamin D levels; however, 48.3%, 4.9%, and 30.1% of patients were deficient, severely deficient, or insufficient, respectively.

### Multiple logistic regression analysis

**Fig. 2:**
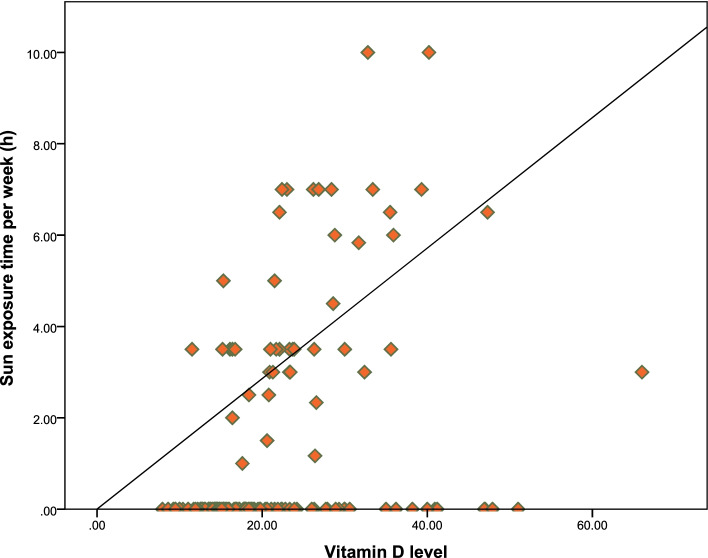
Relation between weekly sun exposure time (h) and serum 25-hydroxyvitamin D level

Table 2 presents the results of multiple logistic regression analysis. A regression model suggested that living in urban settings (AOR, 3.729; 95% CI, 1.134–12.227; *p* = 0.030) and the duration of weekly sun exposure were the two significant predictors of vitamin D deficiency (AOR, 1.653; 95% CI, 1.264 – 2.163; *p* =  < 0.001). Furthermore, Fig. 2 displays the relation between weekly sun exposure time and vitamin D level.

**Table 2:** Multiple logistic regression analysis: predictors of vitamin D deficiency

Variables	Adjusted OR	SE	95% CI	*P*-value
*BMI*	0.980	0.057	0.877—1.095	0.720
*Education*				
Masters and above	0.919	0.953	0.142 – 5.952	0.929
Bachelor	0.672	0.927	0.110—4.147	0.670
Higher secondary school	1.042	0.944	0.164—6.632	0.965
Primary to secondary school	0.716	0.836	0.139—3.683	0.689
No school education	Reference			
*Living location*				
Rural	Reference			
Urban	3.729	0.607	1.134—12.227	**0.030**
*Sun exposure (h/week)*	1.653	0.137	1.264 – 2.163	** < 0.001**
*Serum Calcium level (mg/dl)*	1.441	0.378	0.684—3.024	0.334

Bold *p*-values are significant at a 5% significance level

## Discussion

This study found a high prevalence of vitamin D deficiency among patients with MSDs seeking physiotherapy treatment in hospital settings. A higher prevalence of vitamin D deficiency was observed in the participants with higher BMI, a bachelor’s degree, lower weekly sun exposure time, and lower serum calcium levels and those living in urban settings; however, regression analysis suggested that the weekly duration of sun exposure and living location were the predictors of vitamin D deficiency. We also found no significant differences in vitamin D deficiency among patients with lower back, neck, shoulder, and knee pain and patients with acute, subacute, and chronic pain.

The prevalence observed in our study is similar to that reported in a systematic review on Bangladeshi adults and to global prevalence [17]. The global prevalence of vitamin D deficiency is also similar to the rate we found [22]. Furthermore, consistent with our findings, the prevalence of vitamin D deficiency among patients in India experiencing low back pain was approximately 50% [23]. However, we did not find a significant sex-specific difference in vitamin D deficiency, which is incongruent with the findings of previous studies [17, 24].

Our study found a significant association between BMI and vitamin D deficiency. In line with our findings, previous studies reported that there was a significant decrease in serum vitamin D levels with increasing BMI irrespective of age or sex [25–27]. Our study also revealed that the prevalence of vitamin D deficiency was higher among participants with higher education. In contrast, studies from developed countries found different results [26, 28]. Lifestyles, where vitamin D deficiency depends greatly, may differ country to country. Understandably, in developing countries, such as Bangladesh, participants with higher education mainly engage in office work that forces them to spend longer times indoors [29] and may effectively limit their exposure to direct sunlight than their counterparts who work outdoors. However, additional studies are warranted to validate this conclusion.

The range of vitamin D deficiency may vary from 20 to 90% depending on residential location [14, 30]. We found a significantly high prevalence of vitamin D deficiency among participants living in urban settings compared with that among participants who lived in rural Bangladesh. While Bangladesh is situated in a tropical region that is exposed to more sunlight throughout the year, the scarcity of open spaces in urban areas and differences in the degree of sunlight exposure due to geographical location may explain the impact of residential location [31].

Vitamin D, prominently referred to as sunshine vitamin, has been produced on this earth for more than half a billion years. In the body, 7-dehydrocholesterol absorbs ultraviolet B (UVB) radiation and is converted to previtamin D3, which in turn isomerizes into vitamin D3 when the skin is exposed to sunlight [32]. Previtamin D3 and vitamin D3 also absorb UVB radiation and are converted into a variety of photoproducts, some of which have unique biological properties [32]. Sunlight and vitamin D production have many prehistoric and historical perspectives [32]. A study found that sunlight was a better source of vitamin D than its oral supplementation [33]. However, in our study, the participants had a mean sun exposure duration of only 1.4 h/week. Expectedly, the duration of weekly sun exposure was an independent predictor of vitamin D deficiency among patients with MSDs, indicating the need for direct sun exposure to mitigate vitamin D deficiency in these subjects.

This study has some limitations. First, considering the cross-sectional nature of the present study, a causal relationship between the independent variables and vitamin D status at the time of participation could not be confirmed. Second, data on clothing style, skin color, and sunscreen use would further strengthen the study findings. In the Indian subcontinent, there is ample sunshine throughout the year; however, a study suggested that the percent conversion of 7-dehydrocholesterol to previtamin D and its photoproducts and formation of previtamin D and vitamin D was maximal between 11 a.m. to 2 p.m. during the entire year [34]. In our study, we did not gather information regarding the time of day when the participant was exposed to sun, which may confound our results. Finally, the sample size was relatively small due to a lack of funding and the high cost of vitamin D testing in Bangladesh. Finally, this study was conducted based on Dhaka City; thus, the results should be generalized with caution.

## Conclusions

Our study revealed a high prevalence of vitamin D deficiency among patients with MSDs. To address this issue, patients are recommended to implement simple but vital measures. Along with proper physical exercise to maintain a healthy BMI, patients should adhere to a healthy diet that contains calcium and vitamin D. Additionally, patients with MSDs are advised to have regular sun exposure, preferably from 11 a.m. to 2 p.m., to minimize the risk of vitamin D deficiency. Physicians should also advise vitamin D supplementation for patients with MSDs in addition to other treatments to reduce vitamin D deficiency when required.

## Data Availability

Data will be available from the corresponding author upon reasonable request.

## Abbreviations

MSDs: Musculoskeletal disorders
BMI: Body mass index
AORs: Adjusted odds ratios
SD: Standard deviation
CI: Confidence interval
25(OH)D: 25-Hydroxyvitamin D
